# Accurate vibrational hydrogen-bond shift predictions with multicomponent DFT†

**DOI:** 10.1039/d5sc02165k

**Published:** 2025-05-20

**Authors:** Martí Gimferrer, Lukas Hasecke, Margarethe Bödecker, Ricardo A. Mata

**Affiliations:** a Institut für Physikalische Chemie, Georg-August Universität Göttingen Tammannstraße 6 37077 Göttingen Germany rmata@gwdg.de +49-551-3923149

## Abstract

In this work we explore the use of multicomponent methods for the computational simulation of anharmonic OH vibrational shifts. Multicomponent methodologies have become popular over the last years, but still are limited in their application range. However, by enabling the simultaneous quantum treatment of protonic and electronic wave functions/densities, they hold promise for the treatment of anharmonic effects and proton vibrations in general. This potential has only been probed but not fully realized so far. This study investigates the performance of Nuclear-Electronic Orbital Density Functional Theory (NEO-DFT) in the prediction of water OH shifts upon complexation with organic molecules. We make use of the HyDRA database, expanded to 35 hydrogen-bonded monohydrates of small organic molecules, and evaluate a range of DFT functionals, both hybrid and double-hybrid. We introduce a robust prediction strategy based on common ingredients available when running conventional DFT and NEO-DFT calculations, which for the first time reduces the root mean square deviation (RMSD) values below 10 cm^−1^ for the set. Double-hybrid functionals in combination with a DFT treatment of the proton of interest is found to be particularly promising. The new systems added to the HyDRA dataset are presented and used as an extra test to the methodology.

## Abstract 2

Proton densities from multicomponent methods are used for the computational simulation of anharmonic OH vibrational shifts.

## Graphical abstract

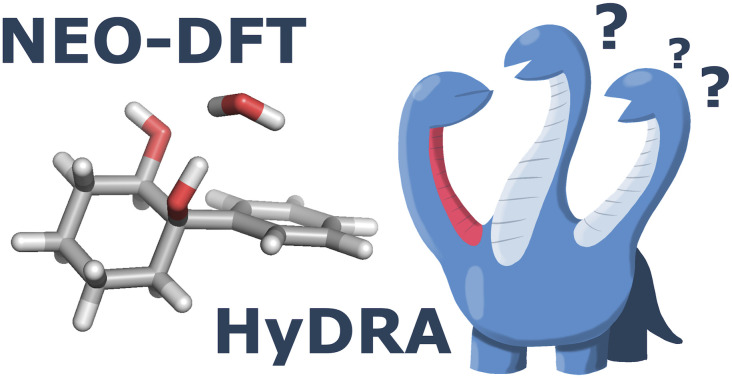

## Introduction

Vibrational spectroscopy is an extremely widespread and popular approach for the study of molecular clusters and interactions in general.^1–3^ With wide applications ranging from interstellar medium^4,5^ to molecular adsorption on surfaces/porous materials,^6,7^ each molecule carries unique signatures that can be tracked through the use of infrared absorption/emission techniques. However, in order to identify said fingerprints and understand how these are influenced by molecular interactions, electronic structure calculations and/or other computational approaches are often times essential.^8–11^

In an attempt to put the predictive power of computational methodologies to the test, two of the authors co-organised a double-blind challenge, focusing on the vibrational spectra of hydrates. The “Hydrate Donor Redshift Anticipation” (HyDRA) challenge presented 10 hydrate test systems to be computed under a time limit.^12,13^ The target observable was the wavenumber downshift of the OH donor band of water, as it forms hydrogen bonds to selected organic molecules. A set of training systems was provided as well, in order for participants to fine-tune or test their methodologies. A total of 20 submissions were registered, showing the strengths and weaknesses of different approaches. In the end, among the top performing methods one could count simple harmonic predictions alongside machine learning protocols. A few lessons could be extracted from the challenge. First of all, the computational effort of the different approaches did not necessarily correlate with the quality of the final results. Particularly in the case of full or partially anharmonic corrected approaches, the results often times deviated significantly from the experimental observations. This was in line with the observations made in a previous challenge on the furan-methanol dimer, whereby anharmonic corrections gave contrasting results.^14,15^ One could also observe that it was a hard task to reach below a 10 cm^−1^ accuracy for the water shifts. In fact, none of the submissions was able to reach a root-mean square deviation (RMSD) below that value.^13^ We expect further improvements in new versions of the challenge, as participants further hone their protocols. But beyond the challenge, the data can be repurposed as a benchmark for the evaluation of new approaches.

We begin by presenting the HyDRA dataset in its first version. It includes both training and test systems used in the challenge, plus additional systems which were collected/submitted since its start. It consists in total of 35 hydrogen-bonded monohydrates of small organic molecules with diverse complexities, and for which experimental redshifts of the symmetric OH stretching vibration have been measured in the jet-cooled gas phase. In this work, we will make use of these experimental data points to benchmark multicomponent DFT. Within the Nuclear-Electronic Orbital Density Functional Theory (NEO-DFT), electrons are treated quantum mechanically with other groups of fermions. Typically, these will be protons of chemical interest. In this case, the hydrogen nuclei are no longer represented by point charges, but instead through Kohn–Sham orbitals. The electronic and protonic subsystems are solved through two separate Fock matrices, coupled through Coulomb interactions as well as electron–proton correlation (if included).^16,17^ We compare a variety of DFT functionals, including both hybrid and double-hybrid types, in conjunction with NEO-DFT. We detail a methodology for predicting vibrational shifts as a function of proton density displacements and compare it to other HyDRA challenge submissions. Our findings demonstrate that, within the NEO-DFT framework, one can effectively and accurately account for anharmonic effects. This capability is essential for the high-accuracy computational evaluation of spectroscopic vibrational shifts, particularly for hydrate systems. Finally, we make use of the newly introduced systems in the dataset to expand our testing of the model.

## Proton charge centroids and level transitions

As mentioned in the previous section, we are interested in establishing correlations between the NEO-DFT position of a quantum proton and the anharmonic corrections to the fundamental OH stretch band. In order to illustrate how the two quantities are related, we will make use of a 1D-Morse potential. The analytic expressions for energies and other expectation values have been provided by Tipping and Ogilvie.^18^ The following discussion draws from the expressions provided by these authors.

**Fig. 1:**
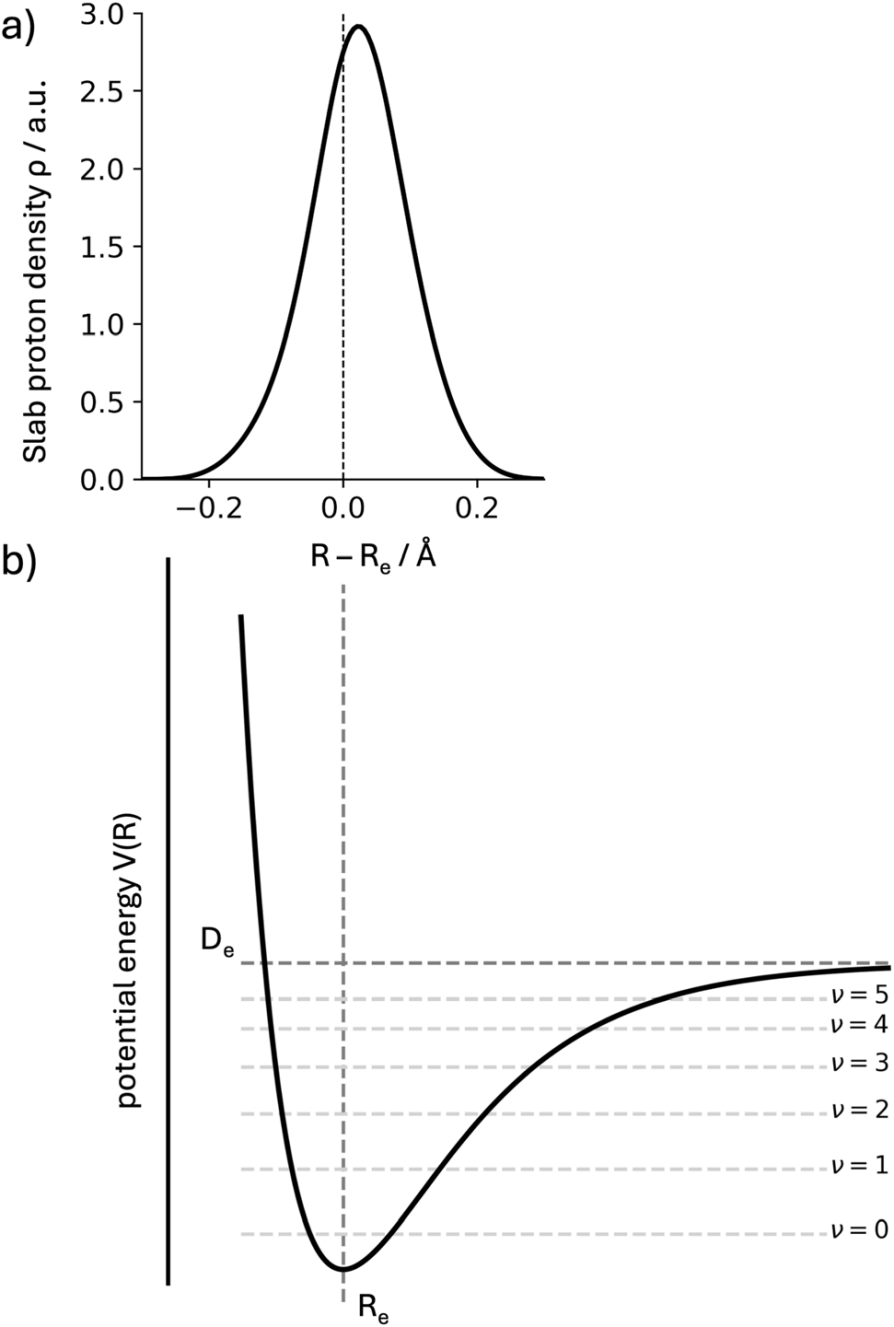
(a) Numerical summation of proton density from a NEO(epc-17.2)-B3LYP calculation in the ACE system. The values are obtained by summing slab grid values (0.01 bohr step sizes) for a fixed position along the bond. The *x*-axis presents this position as the shift from the classical proton position *R*_e_ (positive values are closer to the acceptor atom). (b) 1D Morse potential as defined in eqn (1), with the dissociation energy *D*_e_ and the minimum position *R*_e_ shown. The first states for the Morse oscillator are equally displayed (*ν* ≤ 5). In both cases, the density for the ground state will be shifted away from the minimum *R*_e_ due to the anharmonicity of the potential.

We express the Morse potential with a depth of *D*_e_ and minimum at *R*_e_ (see Fig. 1) as

1 *V*(*R*) = *D*_e_[1 − e^−*a*(*R*−*R*_e_)^]^2^.

The dimensionless anharmonicity parameter *S*^−1^ is defined with2
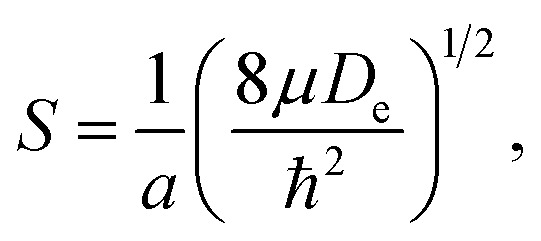
for a Morse oscillator with reduced mass *μ*. It is possible to express the fundamental transition (*ν* = 0 → 1) as well as the density centroid for the oscillator in the ground state (*ν* = 0) on the basis of this anharmonicity parameter. The fundamental transition is given by3
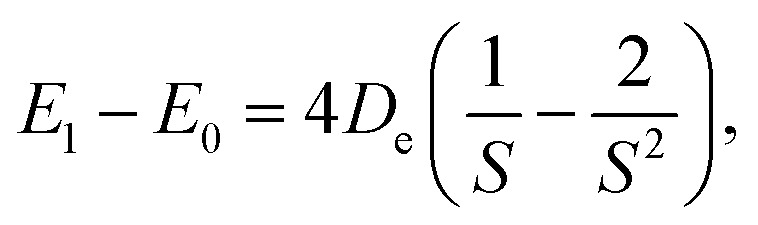
while the position of the density centroid relative to *R*_e_ can be truncated to second order in *S*^−1^4
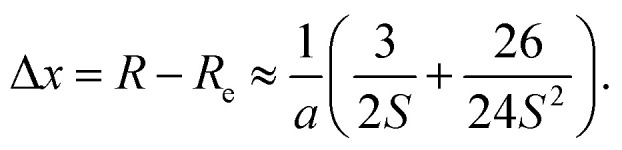


If we compare the two expressions (eqn (3) and (4)) we observe that they scale proportionally to first order. In second order, the trends are opposite (difference in signs for the *S*^−2^ terms). This line of discussion ignores that the depth and shape of the potentials can have a differentiated effect. Whether a linear relationship can be expected will depend on the system under study. But this simple 1D-model already helps to illustrate how the two quantities can be correlated, and why we expect to obtain information about anharmonic corrections to the fundamental OH stretch band through the NEO-DFT centroid. In Fig. 1 we also present the NEO-DFT computed density for one of the systems under study and how it is slightly shifted from the minimum position.

## Introducing the HyDRA dataset

When water forms a hydrogen-bonded dimer with a hydrogen bond acceptor molecule, the symmetry of the symmetric and antisymmetric OH stretching modes is broken. For water in molecular clusters, the two OH stretching vibrations can be described as in-phase and out-of-phase vibrations. The major contribution to the in-phase OH stretching is the stretching of the OH hydrogen-bonded to the acceptor molecule (OH_b_). Because of its high IR activity and its sensitivity to the hydrogen bond strength, the OH_b_ stretching fundamental can provide experimentally well accessible and valuable information about competing conformations in hydrogen-bonded dimers. By subtracting the absolute OH_b_ wavenumber of the molecular cluster from the water monomer symmetric stretching fundamental (3657 cm^−1^),^19^ the hydrogen bond-induced redshifts can be calculated.

**Fig. 2:**
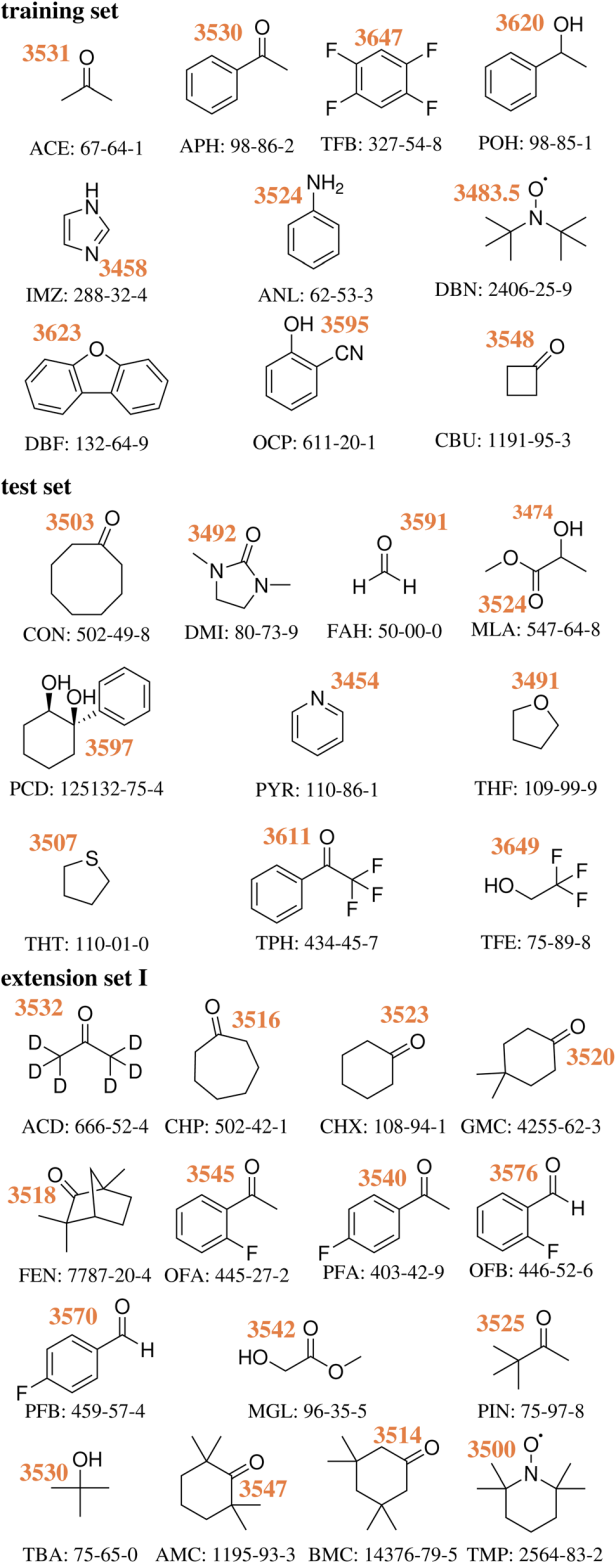
Experimental wavenumbers in cm^−1^ of the hydrogen-bonded OH stretching fundamentals for water in the 35 monohydrates of the training,^12^ test^13^ and first extension set^21^ of the HyDRA database. Structural formulae, CAS registry numbers and acronyms are given for the acceptor molecules. In cases of experimentally observed anharmonic resonances, deperturbed wavenumbers are shown.^22^ Raw wavenumbers are available from ref. 21.

The HyDRA database is a growing experimental database of hydrogen-bonded water OH stretching vibration (OH_b_) wavenumbers in vacuum-isolated monohydrate complexes^20^ at low temperature.^21^ At this point in time, the database contains 35 1 : 1 complexes of water with a small organic molecule (Fig. 2). It was initially created in the context of the first HyDRA blind challenge,^12,13^ in which computational spectroscopy was challenged to blindly predict experimentally measured OH_b_ wavenumbers of 10 test set systems. For this, a training set consisting of 10 hydrogen-bonded monohydrates was provided together with the literature-known and experimentally well-characterized OH_b_ wavenumbers.^12^ The goal was to curate a training and a test set that is diverse in terms of complexation redshifts and different functional groups. Furthermore, in all HyDRA-systems, water is the proton donor in the lowest energy structure. Until the submission of the quantum-chemical predictions, the experimentally measured OH_b_ wavenumbers of the test set systems were kept secret. After the evaluation of the HyDRA blind challenge, the training and test set data were made available.^21^ The database is now extended by 15 additional experimentally unambiguously characterized OH_b_ wavenumbers of hydrogen-bonded monohydrates (extension set I). The hydrogen bond acceptors are: fully deuterated (D6) acetone (ACD),^22^ cycloheptanone (CHP),^22^ cyclohexanone (CHX),^22^ 4,4-dimethylcyclohexanone (GMC),^22^ (−)-fenchone (FEN),^22^ 2′-fluoroacetophenone (OFA),^22^ 4′-fluoroacetophenone (PFA),^23^ 2-fluorobenzaldehyde (OFB),^23^ 4-fluorobenzaldehyde (PFB),^23^ methyl glycolate (MGL),^23^ pinacolone (PIN),^22^*tert*-butyl alcohol (TBA),^23^ 2,2,6,6-tetramethylcyclohexanone (AMC),^24^ 3,3,5,5-tetramethylcyclohexanone (BMC),^22^ and the TEMPO radical (TMP).^24^

The HyDRA database is designed to provide experimental data which allows a straightforward comparison to quantum-chemical calculations. Therefore, the molecular aggregates are measured in a supersonic jet expansion,^25^ in which molecules and molecular clusters are vacuum-isolated and cooled to temperatures below 20 K.^12^ Through this approach, environmental and thermal effects on the conformations and vibrations are minimized. The 35 systems include 33 closed-shell and two open-shell systems (DBN^26^ and TMP^24^). Currently, the hydrogen bond-induced redshifts of the in-phase OH stretching wavenumbers in the database range from 8 cm^−1^ (H_2_O–TFE) to 203 cm^−1^ (H_2_O–PYR).^13^

For many monohydrate clusters, anharmonic resonances^22,24,27^ are observed, when two vibrational states of the same symmetry are in energetic proximity. In this kind of anharmonic resonances, a bright state shares intensity with a dark state, and both states split energetically. Assuming that the dark state has negligible intrinsic intensity, the deperturbed OH_b_ wavenumber can be determined as the intensity centroid of the resonance signals.^22^ In the HyDRA database, anharmonic resonances have been experimentally observed for 11 monohydrated systems: ACE, ACD, APH, CHP, CHX, GMC, FEN, PIN, AMC, BMC and TMP.^21^ In Fig. 2, the deperturbed band positions of the OH_b_ stretching fundamental are shown.

## Computational details

All Kohn–Sham Density Functional Theory (KS-DFT) calculations were performed with the Gaussian16 software (revision A03).^28^ Geometry optimizations were carried out using the hybrid density functionals B3LYP^29^ and PBE0,^30^ and the double-hybrids B2PLYP^31^ and DSD-PBEP86,^32,33^ coupled with the def2-TZVPP full electron basis set.^34^ In all cases, Grimme's D3 dispersion correction with Becke–Johnson (BJ) damping was included in the calculations.^35,36^ For convergence, *tight* thresholds were employed (opt = tight keyword in Gaussian16). Vibrational frequency calculations to confirm zero imaginary frequencies (minima) on the potential energy surface, and to extract the value of the frequency associated with the donor OH stretching, were performed at the same levels of theory.

Initial geometries for optimization for the test and training sets of the HyDRA challenge were extracted from the LS3 submission, the best overall submission that provided geometric (*xyz*) data, except for PCD. For the latter, the LS1 conformer was used. The reason for this choice is discussed in the original paper.^13^ For the newly included systems in the HyDRA database, initial *xyz* coordinates were obtained using a Python script that combines the SMILES molecular representation (extracted from entering the CAS number in PubChem) and RDkit. Then, a water molecule was manually introduced into the system, and conformer analysis was performed with CREST (version 3.0.2) using the sampling of non-covalent complexes and aggregates (NCI mode).^37^ With this, several plausible conformers within a range of 6 kcal mol^−1^ were obtained, reoptimized at the B3LYP/def2-TZVPP level of theory, and ranked by energy (electronic + zero-point vibrational energy, Δ*H*^0^). Conformers with relative energies within 1 kcal mol^−1^ were reoptimized using the 4 different DFT functionals used in the study, selecting as the “best” candidate the lowest in energy by all of them (coincided in all cases).

All single-point NEO-DFT calculations were carried out by employing our NEO program suite implemented in the Molpro 2024.2 package.^38–42^ For the electronic part, the B3LYP functional was employed for all B3LYP and B2PLYP optimized structures and the PBE0 functional for the PBE0 and DSD-PBEP86 ones.^29,30^ In all calculations, and analogously to the geometry optimizations, D3 dispersion correction with Becke–Johnson damping was included.^35,36^ To account for the electronic–nuclear correlation, the epc-17.1 and epc-17.2 functionals were used in this work.^43,44^ In all our multicomponent calculations, the quantum nuclear subsystem includes only the proton involved in the hydrogen-bonded water OH stretching vibration. The basis functions for the quantum proton are placed at the Born–Oppenheimer optimized positions and the expectation value is computed based on the self-consistently obtained nuclear NEO-DFT density. As electronic basis set, the def2-TZVPP basis set together with the def2-QZVPP-JKFIT density fitting basis set were utilized while the PB4-F2 basis set together with the even tempered 10s10p10d10f fitting basis set, with exponents ranging from 
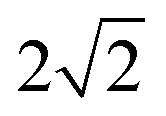
 to 64, was employed as the nuclear basis set.^34,45,46^ Additional calculations were carried out with larger basis sets, namely the def2-QZVPP electronic and PB6-F nuclear basis sets, employing the same density fitting basis sets as mentioned before.^34,46^ We set a threshold of 10^−8^ a.u. for the energy difference within the electronic and nuclear subcycles, the gradient as well as the difference in the density between the individual self-consistent field (SCF) iterations of both subsystems. The global threshold for the absolute NEO energy as sum of all contributions is set to 10^−7^ Hartree. To accelerate the convergence within the SCF subcycles the direct inversion in the iterative subspace (DIIS), starting after the first iteration with a maximum of 10 Fock matrices as basis to extrapolate, is employed.^47,48^ All NEO-DFT calculations utilize the standard grid 3 (SG-3) of Dasgupta and Herbert for numerical integrations.^49^ It should be noted that the NEO-DFT calculation is not the computational bottleneck in our computational protocols. It is faster to run than the analytical Hessian calculation which is needed for the harmonic frequency values.

## Results and discussion

### HyDRA challenge systems

Let us start by revisiting the HyDRA challenge from the perspective of multicomponent DFT methods. To reiterate, the HyDRA challenge involves assessing the accuracy of computational protocols in determining the vibrational shift of the donor OH stretching from vibrational spectroscopy. We will denote this quantity as Δ*ν*_exp_, where 
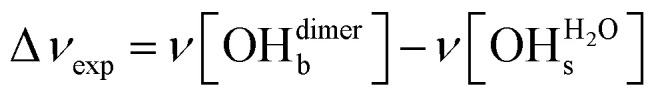
. The value of 
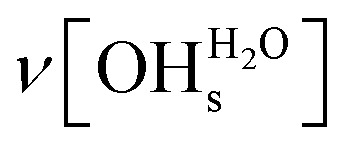
 is determined as 3657 cm^−1^.^19^ The values for the dimers were the deperturbed wavenumbers. The original challenge presented 20 hydrate systems, with 10 (nine of which are closed-shell) forming the training set (with experimental Δ*ν*_exp_ values provided), and the remaining 10 designated as a test set (blind challenge).^12^

Initially, we evaluated the geometry and purely harmonic (PH) shifts, Δ*ω*_harm_, for both training and test sets using four electronic KS-DFT functionals of different nature: two hybrid (B3LYP and PBE0) and two double-hybrid (B2PLYP and DSD-PBEP86). In all cases, dispersion corrections have been included by means of Grimme and coworkers D3 scheme with Becke–Johnson (BJ) damping parameters. For further technical details, we guide the reader to the Computational details section. The raw data for the obtained shifts is gathered in Table S1.† The root mean square deviation (RMSD) values calculated between Δ*ω*_harm_ and Δ*ν*_exp_ were 36.3 cm^−1^ and 52.7 cm^−1^ for the hybrid functionals B3LYP and PBE0, respectively, while the double-hybrids yielded significantly lower values: 24.2 cm^−1^ for B2PLYP and 19.2 cm^−1^ for DSD-PBEP86 (see Table S2†). This improvement in accuracy can be attributed to the incorporation of MP2-like correlation into the functional expressions. It is important to remark that low RMSD values are not expected from PH methods since anharmonicity effects are not accounted for. More accurate results would be mere error compensation, and not physically grounded.

For each system, the numerical discrepancy between Δ*ω*_harm_ and Δ*ν*_exp_ directly reflects the amount of anharmonicity associated with the formed hydrogen bond (H–OH) upon hydration as well as the method error (DFT functional and basis set used). We will argue that double-hybrid functionals should reduce the latter significantly, to the point that the harmonic approximation errors dominate. Multicomponent DFT calculations that treat the H-atom as a quantum particle intrinsically account for both harmonic and anharmonic effects. This is evidenced by the shift/polarization of the density of the quantum hydrogen atom, henceforth proton density, relative to its classical atomic position. This shift, termed Δ*x*^H^, is evaluated by calculating the displacement of the expectation value (centroid) of the proton density (***x***^**H**^) from its classical nuclear position (***R***^**H**^): Δ*x*^H^ = |***x***^**H**^ – ***R***^**H**^|. Subtracting the shift (equally evaluated) from an isolated water molecule to that within the system allows for a direct comparison with the anharmonic effects neglected in conventional DFT calculations:

5 Δ*x*^H^  =  |***x***^**H**^ − ***R***^**H**^| − |***x***^**H,H**_**2**_**O**^ − ***R***^**H,H**_**2**_**O**^|.

**Fig. 3:**
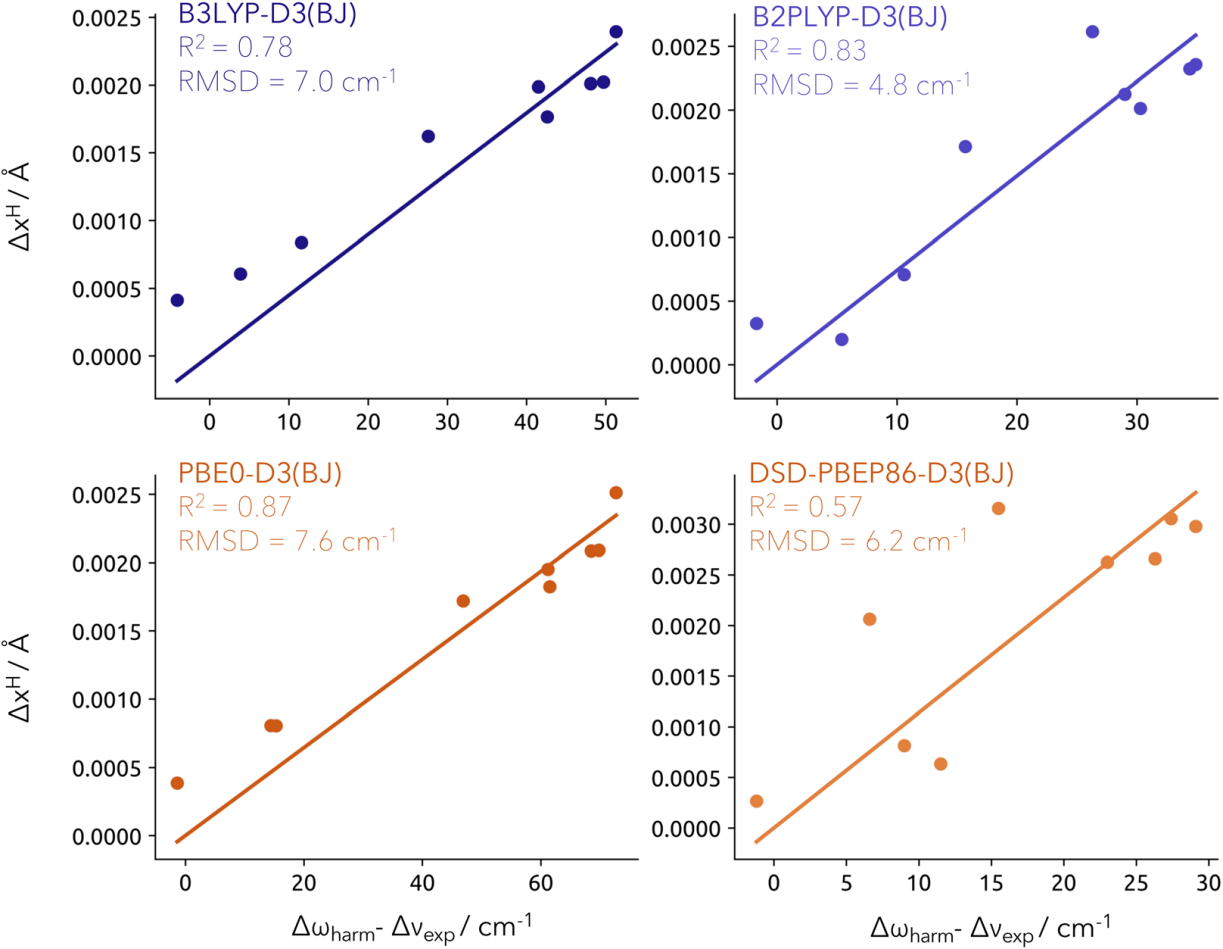
Linear fit for the training set (9 closed-shell systems) constrained to cross the [0, 0] at the different levels of theory. RMSD provided in cm^−1^. Note that the *x*-axis scale changes significantly depending on the method applied, with a much shorter range for double hybrid functionals.

The obtained proton density depends on the choice of the electron–proton correlation functional. The most widely used LDA-type functionals are the epc-17.1 and epc-17.2 functionals developed by Hammes–Schiffer and coworkers.^43,44^ Those were recently benchmarked by Yang *et al.*^50^ by calculating vibrational spectra from constrained NEO molecular dynamics. It was found that the epc-17.1 functional produces results similar to Born–Oppenheimer DFT while the epc-17.2 functional or NEO-DFT without any electron–proton correlation reproduce the main features of the experimental spectra. For our work, we compare the difference in the obtained expectation values of both epc functionals and without electron–proton correlation in Fig. S1† for the training and test sets. In line with the results of Yang *et al.*, we find that the results obtained with epc-17.1 are rather featureless and somewhat close to the Born–Oppenheimer proton positions. The epc-17.2 results exhibit larger deviations from the classical positions and a robust correlation with the frequency shifts. A similar distribution is found if no electron–proton correlation is invoked within the NEO-DFT method. However, even though the centroid values are largely unaffected by removing electron–proton correlation, the overall nuclear density does change severely as shown by Tao *et al.*^51^ Therefore, we restrict ourselves in this work to the use of the epc-17.2 electron–proton correlation functional. In Fig. 3, we illustrate the linear correlation between Δ*x*^H^ (*y*-axis) derived from NEO-DFT calculations (for details, see Computational details section) and the Δ*ω*_harm_ − Δ*ν*_exp_ values (*x*-axis) calculated at the different levels of theory. Importantly, the linear regressions have been constrained to intercept the origin (0, 0). This is the reference value for the isolated water molecule, in order to keep consistency with the quantity definition (eqn (5)). This fitting strategy allows for the prediction of Δ*ω*_harm_ − Δ*ν*_exp_ values for any given system and subsequently provides a correction to the harmonic wavenumbers obtained at the associated conventional DFT level. The resulting model will be referred to as Model-9 (9 training points).

Applying our model to the training set yielded RMSD values lower than 10 cm^−1^, regardless of the functional employed. Among them, B2PLYP resulted in the lowest RMSD with a value of 4.8 cm^−1^. Interestingly, DSD-PBEP86 also provided relatively low errors despite presenting a modest *R*^2^ of 0.57. This is attributed to overall lower Δ*ω*_harm_ − Δ*ν*_exp_ values compared to those derived from other functionals. As previously mentioned, the difference between the harmonic computed values and the experimental shifts cannot be solely attributed to anharmonic effects. It also carries the deficiencies of the level of theory chosen for the harmonic prediction. From the assumption that double-hybrid functionals are more robust in the description of the potential energy surface, it makes sense that our model correction is best carried by B2PLYP-D3(BJ) and DSD-PBEP86-D3(BJ).

When Model-9 is used to predict Δ*ω*_harm_ − Δ*ν*_exp_ values for the test set, the RMSD values are 12.7 cm^−1^ (B3LYP), 12.8 cm^−1^ (PBE0), 7.2 cm^−1^ (B2PLYP) and 6.8 cm^−1^ (DSD-PBEP86). This is in extremely close agreement with the experiment. All quantities used to evaluate the RMSDs are collected in Table S1.† It should be noted that the position of the quantum proton is determined through a self-consistent field NEO-DFT calculation, no optimization procedure is used. In order to verify whether or not this position is influenced by our choice of protonic basis set, all calculations were repeated with an even larger protonic (PB6-F) as well as electronic (def2-QZVPP) basis set.^46^ The results indicate no quantitative difference as shown in Tab. S3.

To assess the performance of our model in further detail, we present in Fig. S2† the system-specific prediction errors for Δ*ω*_harm_ − Δ*ν*_exp_, comparing these results to the best purely harmonic submission (PH5) from the HyDRA challenge. The error is defined as the deviation to the experimental value. Overall, the NEO-based model displays impressive performance given that the errors (in absolute value) for the double-hybrids are generally smaller than *ca.* 10 cm^−1^ (see Table S4†). The most problematic systems are DMI, FAH and THF, with a maximum absolute deviation of 13 cm^−1^.

**Fig. 4:**
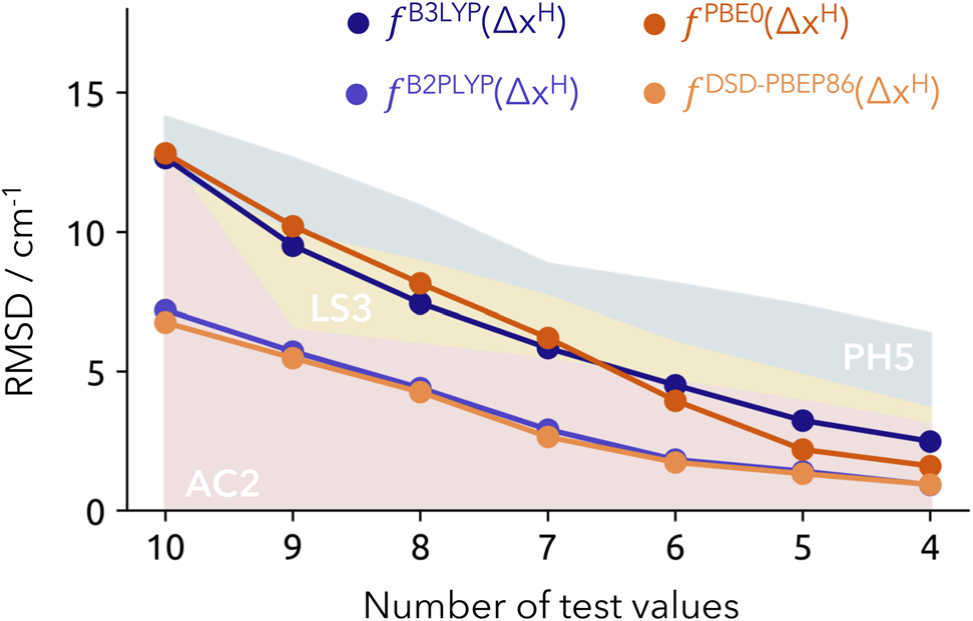
Overall RMSD from the NEO-based model for the test set (Model-9). Each unit decreased in the number of test values (*x*-axis) involves removing the “worst” predicted value. Reference values from other submissions are represented as colored surfaces: AC2 (red), LS3 (yellow) and PH5 (blue). RMSD provided in cm^−1^.

In order to compare to the best submissions of the HyDRA challenge, we opt for a similar diagram as used in the original publication. We provide incremental RMSD values derived from removing the worst prediction of the test set (see Fig. 4), and contrasting them with the results of the best-performing methods from the HyDRA challenge: PH5, AC2 and LS3.^13^ The trends for the PH5 and our model results (with double hybrids) are notably similar, with the latter showing better accuracy by approximately 5 cm^−1^. It also displays better performance to that of the learning strategy (LS) submissions like LS3, and the anharmonically corrected protocols like AC2 – the top-performing methods within the HyDRA challenge with RMSD values graphically represented as yellow and red surfaces in Fig. 4, respectively.^13^ A significant advantage of our proposed model over learning strategies lies in its simplicity, resulting from the direct calculation of the protonic density. This confirms the robustness of our model with the double hybrid functionals in producing reliable and accurate results for the test set, even when using a limited number of fitting points (9 in total). The caveat that should of course be mentioned is that we are not submitting our estimates blindly. We profit from the structural sampling already performed by the other groups and it is unfair to generally compare to blind challenge conditions. Nonetheless, it is reassuring to observe that the deviations in the training set are comparable to that of the test.

It is worth noting that for the hybrid GGA functionals B3LYP and PBE0, removing the constraint from the linear regressions yields improved fittings (according to the statistical *R*^2^ indicator) for the training set. However, this revised model, yields less satisfactory results when applied to the test set (see Table S5†), a clear sign of over-fitting. Furthermore, we believe the physical interpretation of calculating vibrational shifts is compromised with such a choice.

### Extended HyDRA set

We will now address the prediction of vibrational shifts for an additional suite of 14 new systems included in the HyDRA database. The dimer with the TEMPO radical is left out, as we are only considering closed-shell systems. We again employ our model, but a new linear fit is constructed using the data from both training and test sets (Model-19, 19 systems/data points). The resulting linear fit is depicted in Fig. S3,† revealing similar *R*^2^ and RMSD values as those achieved using only the training set (Fig. 3). This improvement serves to enhance the model's robustness for future predictions, despite any minor declines in statistical metrics like *R*^2^ or RMSD. However, it should be noted that the linear fits are only slightly changed from Model-9 (fit with training systems) to Model-19 (fit with training + test systems), see Table S7.†

**Fig. 5:**
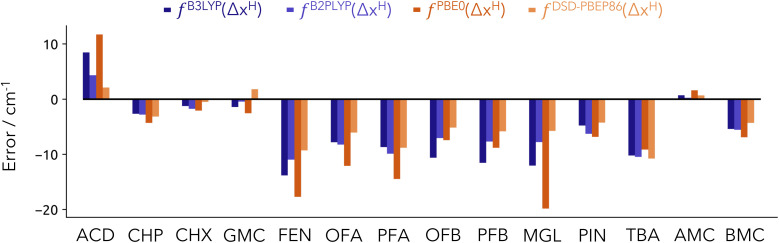
Error in the predictions using Model-19 for the 14 extra molecular systems (extension set I) included in the HyDRA database. Errors provided in cm^−1^.

In Fig. 5, we show the prediction error associated with the NEO-based model for these additional systems. As reference values of performance from other methodologies are unavailable, we focus exclusively on the accuracy of our predictions. Consistent with the test set analysis (Fig. S2†), the double hybrid functionals surpass the performance of hybrid functionals. The new Model-19 does demonstrate improved performance with B3LYP, approaching the accuracy of double hybrids. PBE0 consistently yields the least favourable results, regardless of the number of points used for training. In the case of B2PLYP, the largest error arises for the FEN hydrate system with a value of *ca.* 11 cm^−1^ (see Table S6†). Given the complexities of the additional systems, we conclude that Model-19 provides satisfactory results. The corresponding fitting parameters for Model-19 are given in Table S7.

**Fig. 6:**
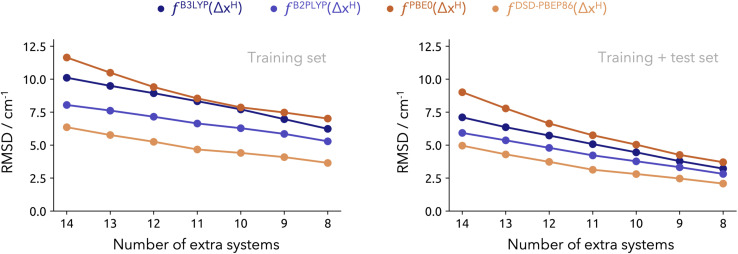
Overall RMSD for the extension set I from Model-9 (left) Model-19 (right) points. Each unit decreased in the number of test values (*x*-axis) involves removing the “worst” predicted value. RMSD provided in cm^−1^.

Lastly, in Fig. 6 we present the incremental RMSD values from both Model-9 and Model-19, on the extension set I. Interestingly, all functionals exhibit a similar trend. The DSD-PBEP86 and B2PLYP methods yield the lowest prediction errors, ranging from approximately 6 cm^−1^ across all 14 systems down to around 3 cm^−1^ when considering only 8 systems (in both cases). The accuracy of Model-19 is impressive, overperforming Model-9 in all accounts. However, these are improvements in the range of just a few reciprocal centimeters. There is not a lot of room for improvement, as one approaches the experimental uncertainty (around 2 cm^−1^).

Overall, the NEO-based fitting model introduced in this study demonstrated remarkable performance in combination with B2PLYP and DSD-PBEP86. The results not only showcase a high degree of accuracy for the majority of the systems but also exhibit consistent robustness across the additional 14 hydrated systems integrated into the HyDRA database. Even with a very small number of training points, Model-9 was able to deliver predictions with RMSD below 10 cm^−1^ for 24 hydrate dimers.

## Conclusions

In this work we present and make use of a first iteration of the HyDRA dataset, a collection of experimentally measured red shifts of the in-phase water OH stretching fundamental upon complexation. The latter is comprised of 35 hydrogen-bonded (hydrated) dimers, building upon the data of the homonymous challenge. In our calculations, we evaluated various DFT functionals in conjunction with NEO-DFT. In line with other benchmarks in the literature, double-hybrid harmonic calculations outperform hybrid functionals, albeit exhibiting similar trends. We propose a straightforward scheme to estimate the anharmonicity of the bonds based on multicomponent theory. The NEO-DFT position of the quantum proton of interest was used as a measure. We established a linear relationship between the shift of the quantum proton relative to its Born–Oppenheimer optimized position. Different fits were used for different combinations of electron–electron and electron–proton correlation functionals. In all cases, the linear correction obtained in this form dramatically improved the agreement between computational and experimental values. This is also, to the best of our knowledge, the first time that a computational protocol offers an accuracy below 10 cm^−1^ for the HyDRA test set (comprised of 10 systems).

The best obtained models, just as with the harmonic predictions, were derived from the two double hybrid functionals featured in the study (B2PLYP and DSD-PBEP86). The correction also compensates for some model deficiencies, but it appears to perform best when the latter is reduced. Anharmonicity appears to be well-captured, with the quality of the fit depending only slightly on the specifics of the fit (*e.g.*, whether or not the intercept is kept fixed) or on the number of points used for training (the results of Model-9 and Model-19 are rather similar, see ESI†). The good performance with the HyDRA test set was repeated for an extended set of 14 hydrate dimers.

These findings highlight the utility of multicomponent DFT as a critical tool in computational vibrational spectroscopy. After all, one single-point calculation proves sufficient to quantify the anharmonicity of an hydrogen bond, based on these results. The study was constructed solely on reference experimental data, doing without high-level vibrational anharmonic calculations for testing. For the systems sizes featured these would either not be feasible or even reliable. As demonstrated in our previous challenges,^12–15^ the blind computational prediction of wavenumbers or their shifts does not approach vibrational benchmark standards, error bars close to the spectroscopy accuracy of 1 cm^−1^.

In the future we will explore if such relations still hold for molecules other than water, and how far such approaches can be used in cases where mode coupling is observed. The OH bond remains a staple of spectroscopy and chemical analytics. The potential for multicomponent methods to be applied in the routine characterisation of these bonds appears promising.

## Author contributions

M. G.: data curation, formal analysis, writing – original draft, writing – review & editing. L. H.: data curation, formal analysis, writing – original draft, writing – review & editing. M. B.: writing – review & editing. R. M.: conceptualization, funding acquisition, writing – original draft, writing – review & editing.

## Conflicts of interest

There are no conflicts to declare.

## Supplementary Material

SC-016-D5SC02165K-s001

## Data Availability

ESI† available: shifts of the vibrational wavenumber associated with the symmetric stretching for each functional including the experimental shifts (Table S1†), harmonic RMSDs on the HyDRA database for each functional (Table S2†), performance of Model-9 utilizing the def2-QZVPP/PB6-F basis sets (Table S3†), individual errors on the test set obtained with Model-9 (Table S4†), performance comparison of the constrained and unconstrained ansatz of Model-9 on the HyDRA test set (Table S5†), individual errors on the extension set I obtained with Model-19 (Table S6†), fitting parameters of Model-9 and Model-19 (Table S7†), data distribution for the training and test set computed with different electron–proton correlation functionals (epc-17.1, epc-17.2) and without electron–proton correlation (Fig. S1†), errors of the test set systems obtained with Model-9 in comparison to submission PH5 of the HyDRA challenge (Fig. S2†), linear fit performance of Model-19 on the training and test set (Fig. S3†). Optimized geometries (*xyz* files) and all inputs needed to reproduce the calculations reported, together with their associated output files, can be found in GRO.DATA (https://doi.org/10.25625/UZOMEV).
