# Supplementary material for: Accurate vibrational hydrogen-bond shift predictions with multicomponent DFT
Source: Chem Sci. 2025 May 20;16(24):11002–11. doi: 10.1039/d5sc02165k (PMC12096501; doi:10.1039/d5sc02165k)
Supplement: SC-016-D5SC02165K-s001 [file SC-016-D5SC02165K-s001.pdf]

Supplementary Information (SI) for Chemical Science.
This journal is © The Royal Society of Chemistry 2025

Table S 1 Shifts (in cm^{-1}) of the vibrational wavenumber associated with the symmetric stretching for each functional included in the study, and the deperturbed experimental value.

Set	Mol.	Exp.	B3LYP-D3BJ	PBE0-D3BJ	B2PLYP-D3BJ	DSD-PBEP86-D3BJ
	H_2O	0	0.0	0.0	0.0	0.0
TRAINING	ACE	126	174.1	195.8	160.4	155.1
	APH	127	176.7	195.5	161.9	154.4
	TFB	10	5.9	8.6	8.3	8.8
	POH	37	40.9	52.3	42.4	48.5
	IMZ	199	250.3	271.7	225.3	214.5
	ANL	133	160.6	179.9	148.7	139.6
	DBF	34	45.6	48.4	44.6	43.0
	OCP	62	103.5	123.2	91.0	85.0
	CBU	109	151.6	170.5	139.3	135.3
TEST	CON	154	206.1	228.7	189.9	183.4
	DMI	165	207.3	225.8	190.6	183.6
	FAH	66	103.9	119.5	97.6	95.0
	MLA	133	188.2	218.2	170.9	164.9
	PCD	60	56.6	77.3	59.9	66.2
	PYR	203	265.1	285.4	237.7	225.5
	THF	166	212.4	226.6	204.7	199.8
	THT	150	205.4	229.8	180.3	173.9
	TPH	46	80.4	90.3	71.1	65.9
	TFE	8	9.8	13.3	11.7	12.1
EXTENSION I	ACD	125	174.2	195.9	160.5	155.1
	CHP	141	193.7	215.7	179.4	173.8
	CHX	134	184.1	204.9	169.9	163.5
	GMC	137	187.3	208.6	171.3	164.5
	FEN	139	194.8	215.0	178.8	172.5
	OFA	112	161.8	180.4	148.0	141.2
	PFA	117	172.8	192.3	158.0	150.6
	OFB	81	135.4	147.7	119.2	110.5
	PFB	87	143.7	157.6	127.0	118.2
	MGL	115	174.5	201.9	155.8	148.9
	PIN	132	181.6	203.0	167.1	161.0
	TBA	127	180.1	197.5	167.9	163.9
	AMC	110	160.0	179.3	145.2	138.6
	BMC	143	195.8	216.5	181.4	174.3

Table S 2 RMSDs on the HyDRA database (separated by sets) in cm^{-1} for each functional included in the study.

	B3LYP-D3BJ	PBE0-D3BJ	B2PLYP-D3BJ	DSD-PBEP86-D3BJ
TRAINING	36.3	52.7	24.2	19.2
TEST	43.9	62.1	29.4	24.0
EXTENSION I	52.9	72.7	37.9	31.3
TRAINING + TEST	40.5	57.8	27.0	21.8
ALL	46.2	64.6	32.1	26.3

Table S 3 Comparison of R^2 values, the RMSDs of the linear fits and the RMSDs on the HyDRA test set obtained with the fit of training set in cm^{-1} for the each individual functional obtained with the def2-QZVPP electronic basis set and PB6-F nuclear basis set.

	B3LYP-D3BJ	PBE0-D3BJ	B2PLYP-D3BJ	DSD-PBEP86-D3BJ
		R^2		
Training set	0.92	0.93	0.82	0.64
Test set	0.64	0.77	0.75	0.64
All	0.77	0.85	0.79	0.64
		Fit RMSD / cm^{-1}		
Training set	5.5	7.1	6.3	6.8
Test set	12.3	13.0	7.3	6.6
All	9.6	10.7	7.0	6.8
		HyDRA RMSD / cm^{-1}		
Test set	12.6	13.5	7.6	6.7

Table S 4 Overview of the individual errors on the test set in cm^{-1} obtained by employing Model-9.

Test system	B3LYP-D3BJ	PBE0-D3BJ	B2PLYP-D3BJ	DSD-PBEP86-D3BJ
CON	-3.18	-2.83	-1.16	0.21
DMI	11.12	15.83	9.9	10.36
FAH	-6.2	-9.87	-10.93	-11.83
MLA	3.63	-2.02	2.02	1.28
PCD	25.09	18.46	6.8	2.31
PYR	-9.42	-4.49	0.98	6.01
TFE	13.85	14.36	3.19	-0.84
THF	-4.01	2.89	-13.13	-10.0
THT	-17.75	-23.3	-1.06	3.34
TPH	-4.01	-1.54	-3.48	-2.8

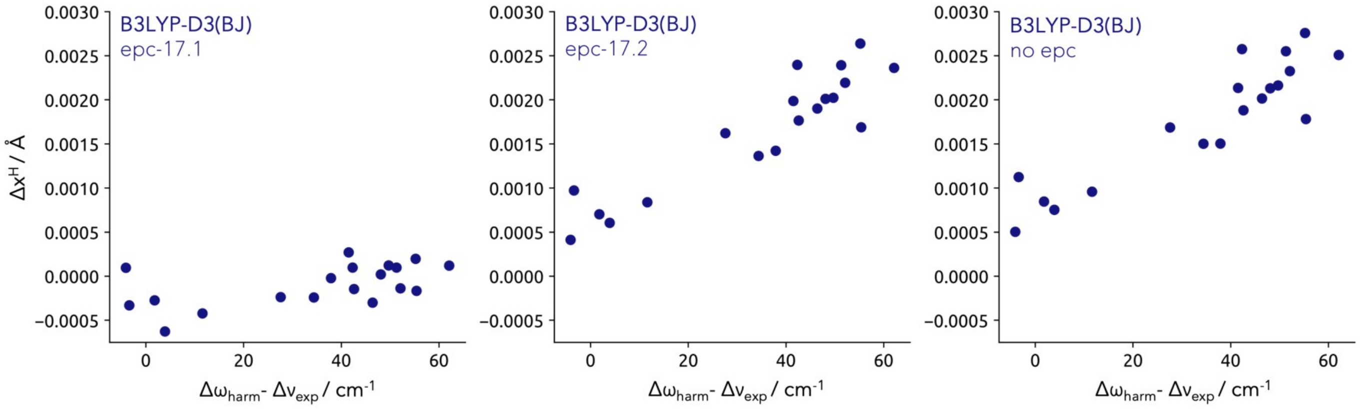

Figure S 1 Comparison of the obtained data distribution for the training and test set computed with different electron-proton correlation functionals (epc-17.1 on the left and epc-17.2 in the middle) together with results employing no epc functional (on the right), all utilizing the electronic B3LYP-D3(BJ) functional.

Table S5 Comparison of R^2 values, the RMSDs of the linear fits and the RMSDs on the HyDRA test set obtained with the fit of training set (Model-9) in cm^{-1} for the constrained and unconstrained intercept for each individual functional.

	B3LYP-D3BJ	PBE0-D3BJ	B2PLYP-D3BJ	DSD-PBEP86-D3BJ
**Unconstrained fit**				
R^2				
Training set	0.97	0.97	0.86	0.65
Test set	0.72	0.79	0.73	0.60
All	0.84	0.89	0.80	0.64
Fit RMSD / cm^{-1}				
Training set	3.8	4.5	5.1	7.3
Test set	13.1	13.2	8.0	7.9
All	9.3	9.7	6.7	7.7
HyDRA RMSD / cm^{-1}				
Test set	12.1	13.3	7.7	8.8
**Constrained fit**				
R^2				
Training set	0.78	0.87	0.83	0.57
Test set	0.31	0.65	0.66	0.55
All	0.56	0.77	0.75	0.57
Fit RMSD / cm^{-1}				
Training set	7.0	7.6	4.8	6.2
Test set	12.5	12.4	7.0	6.6
All	10.3	10.4	6.1	6.5
HyDRA RMSD / cm^{-1}				
Test set	12.7	12.8	7.2	6.8

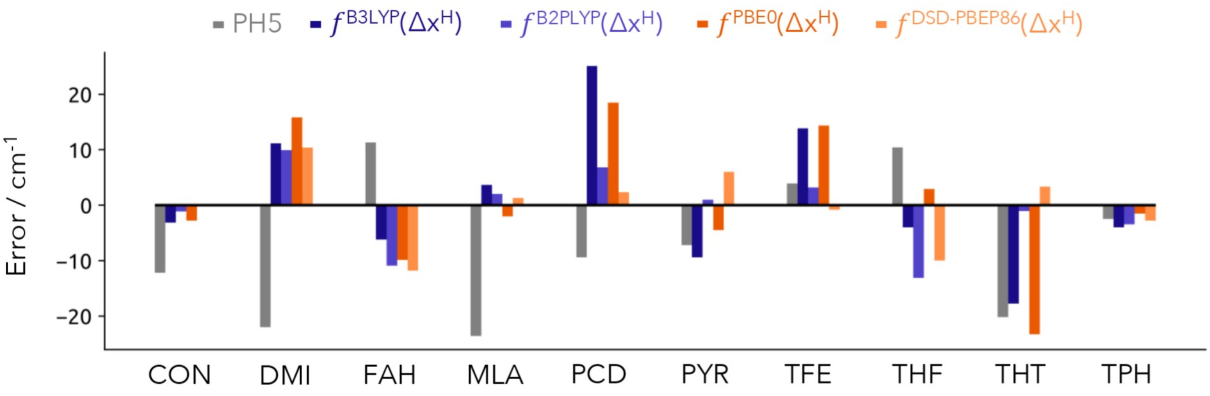

Figure S 2 Error in the predictions using Model-9 for the test set molecular systems included in the HyDRA database in comparison with the original PH5 submission. Errors provided in cm^{-1}.

Table S 6 Overview of the individual errors on the extension set I in cm^{-1} obtained by employing Model-19.

Extra system	B3LYP-D3BJ	PBE0-D3BJ	B2PLYP-D3BJ	DSD-PBEP86-D3BJ
ACD	8.45	11.73	4.33	2.1
CHP	-2.65	-4.31	-2.82	-3.15
CHX	-1.25	-2.09	-1.77	-0.52
GMC	-1.42	-2.57	-0.45	1.79
FEN	-13.84	-17.71	-10.96	-9.3
OFA	-7.83	-12.09	-8.24	-6.06
PFA	-8.7	-14.48	-9.89	-8.83
OFB	-10.63	-7.45	-7.06	-5.17
PFB	-11.54	-8.82	-7.69	-5.85
MGL	-12.04	-19.83	-7.79	-5.78
PIN	-4.8	-6.83	-6.29	-4.25
TBA	-10.23	-9.14	-10.48	-10.78
AMC	0.69	1.6	0.24	0.68
BMC	-5.41	-6.92	-5.56	-4.32

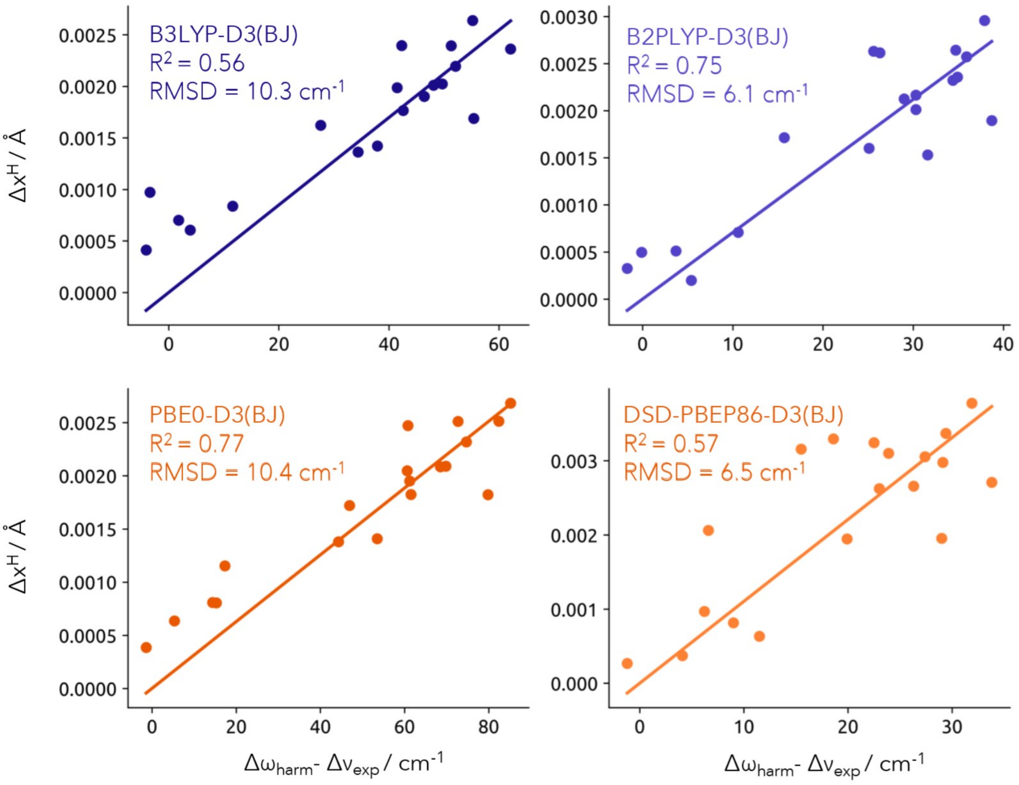

Figure S 3 Linear fit for the training and test sets (Model-19) constrained to cross the [0, 0] at the different levels of theory. RMSD provided in cm^{-1}.

Table S7 Obtained fitting parameters of the linear regression performed on the training set and combined test and training sets as $\Delta x^{H} = a \cdot (\Delta\omega_{harm} - \Delta\nu_{exp}) + b$ (Model-9 and Model-19, respectively) with and without the intercept constrained to the origin.

	fixed intercept [0, 0]	unconstrained	
	a / 10^{-5} Å cm	a / 10^{-5} Å cm	b / Å
**Model-9**			
B3LYP	4.484	3.284	0.0005
PBE0	3.226	2.526	0.0003
B2PLYP	7.409	6.445	0.0004
DSD-PBEP86	11.378	8.687	0.0006
**Model-19**			
B3LYP	4.242	2.836	0.018
PBE0	3.141	2.372	0.017
B2PLYP	7.068	5.856	0.019
DSD-PBEP86	11.037	8.59	0.017
